# Field trials of chemical suppression of embryonic cane toads (*Rhinella marina*) by older conspecifics

**DOI:** 10.1002/ece3.6678

**Published:** 2020-08-31

**Authors:** Samantha McCann, Michael Crossland, Matthew Greenlees, Richard Shine

**Affiliations:** ^1^ School of Life and Environmental Sciences University of Sydney Sydney NSW Australia; ^2^ Department of Biological Sciences Macquarie University Sydney NSW Australia

**Keywords:** anura, biocontrol, *Bufo marinus*, chemical cues, invasive species, pheromones, tadpoles

## Abstract

1. Laboratory experiments have shown that the viability of embryos of the invasive cane toad (*Rhinella marina*) can be reduced by exposure to chemical cues from older conspecific larvae. These effects (very strong in laboratory trials) may offer an exciting new approach to controlling this problematic invasive species in Australia. However, the degree to which the method works in natural environments has yet to be assessed.
2. Our experiments in the laboratory and in seminatural outdoor waterbodies show that chemical cues from tadpoles do indeed suppress the growth, development, and survival of conspecific larvae that are exposed as embryos and do so in a dose‐dependent manner; higher tadpole densities cause greater suppression of embryos.
3. In seminatural outdoor waterbodies, suppressor‐exposed tadpoles were less than half as likely to survive to metamorphosis as were controls, and were much smaller when they did so and hence, less likely to survive the metamorph stage. Additionally, female cane toads were less likely to oviposit in a waterbody containing free‐ranging (but not cage‐enclosed) tadpoles, suggesting that the presence of tadpoles (rather than the chemical cues they produce) may discourage oviposition.
4. Broadly, our results suggest that the suppression effect documented in laboratory studies does indeed occur in the field also, and hence that we may be able to translate that approach to develop new and more effective ways to reduce rates of recruitment of peri‐urban populations of cane toads in their invasive range.

## Graphical abstract

When cane toad eggs are exposed to cane toad tadpoles during development, the eggs often experience stunted development and do not produce viable tadpoles. While adult toads will avoid laying their eggs in ponds containing toad tadpoles, this behavior does not occur if the tadpoles are contained within a small net. This is encouraging for the control of cane toads in Australia as it suggests that adult toads can be manipulated into laying their eggs into ponds where their clutch will not develop into successful tadpoles.

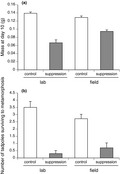

## 1 INTRODUCTION

Results from laboratory studies often do not translate effectively to natural conditions (are not “externally valid”: Brænd, Klovning, & Straand, 2017; Flowe, Finklea, & Ebbesen, 2009; Hansen & Jones, 2017). In ecological research, laboratory studies often provide straightforward results with high effect sizes, whereas attempts to repeat those studies at a larger scale in the natural environment are weakened by confounding variables such as fluctuations in biotic and abiotic conditions, genetic diversity among the study organisms, and interactions from other taxa that are pathogens, parasites, predators, or competitors of the main study species (Diamond, 1983). To translate laboratory‐derived results into the field, the first step is to apply laboratory protocols in a seminatural setting—thereby introducing important parts of the complexity that occurs in the environment while retaining the power of factorial hypothesis‐testing experimental design. Only after we understand external influences on the effectiveness of a new protocol can we cost‐effectively expand the application of that method (if successful) to landscape‐scale trials.

These difficulties in translating research from the laboratory to the field are especially problematic when the aim of the work is intrinsically challenging. The control of invasive species falls firmly into that category, because by definition invasive species are those that thrive in the locations to which they have been translocated, and often achieve population densities far higher than in the natural range (e.g., Lampo & Bayliss, 1996). Successful invaders often are highly fecund and ecologically flexible, exacerbating the challenges of control (Allen, Street, & Capellini, 2017). Although a few biocontrol programs have had spectacular success, the vast majority have failed to translate effectively from the laboratory to the field (Saunders, Cooke, McColl, Shine, & Peacock, 2010). One major impediment has been a failure to understand the ecology of the system in sufficient detail before applying control measures (Pluess et al., 2012).

Cane toads (*Rhinella marina*, formerly *Bufo marinus*) are large (exceptionally, to >1 kg) bufonid anurans native to Latin America. Toads were brought to Queensland, Australia, in 1935 to control insect pests of commercial agriculture, and have since spread across much of the tropics and subtropics of that continent, causing population declines in several species of native fauna (Lever, 2001; Shine, 2010). Many Australian frog‐eating predators lack physiological resistance to the toads' powerful defensive toxins, due to the absence of native bufonids in Australia. Thus, as toads have spread, many native predators have been killed (Shine, 2010). Unfortunately attempts to reduce numbers of cane toads have been largely unsuccessful (see Tingley et al., 2017), and toads currently are found over 1.2 million km^2^ of northern and eastern Australia (Urban, Phillips, Skelly, & Shine, 2008) at much higher densities than in their native range (Lampo & Bayliss, 1996). As the toads continue to spread, we urgently need to develop effective management strategies in order to reduce the impact of toads on native fauna.

Research on early (aquatic) life stages of cane toads has revealed high rates of cannibalism of eggs and hatchlings by older tadpoles (Crossland, Hearnden, Pizzatto, Alford, & Shine, 2011), and a species‐specific waterborne chemical that suppresses the growth of embryonic cane toads if they are exposed during early larval life (“the suppression cue”: Clarke, Crossland, Shilton, & Shine, 2015; Clarke, Crossland, & Shine, 2016; Crossland & Shine, 2011). Under laboratory conditions, cue‐exposed embryos develop into tadpoles with reduced rates of growth, development, and survival (Clarke et al., 2015, 2016). This novel approach to toad control, if transferrable to field conditions, suggests that we could reduce recruitment of cane toads from a pond by adding live toad tadpoles (in mesh cages), or even just the suppression cue itself. Current knowledge of the suppression cue is based almost entirely on laboratory research. The usual protocol has been to expose the developmental stage immediately posthatching (hereafter, “hatchling”, Gosner stage 18) to cues. At this stage, the larva is free from the gelatinous egg‐string but is not yet capable of swimming (Gosner, 1960). These trials have determined that the cue is chemical and not a biological organism, remains effective after being frozen but not after being dried, and can be diluted to concentrations of approximately 0.004 tadpoles per liter without losing its suppressive capability (Clarke et al., 2016). The suppression cue does not influence the growth or viability of tadpoles of a range of native frog species from tropical Australia, suggesting that deployment of the cue is unlikely to cause collateral damage to native fauna (Clarke et al., 2016). The only published attempt to test the suppression cue under natural conditions was a small trial by Clarke et al. (2015), who reported suppression of growth but not survival in embryos (*N* = 15 experimental, 15 control) placed in mesh cages within a natural pond containing toad tadpoles compared to a pond without toad tadpoles.

The current paper translates this laboratory‐based research to small, seminatural waterbodies, to evaluate the effectiveness of the suppression cue in decreasing the viability of cane toad tadpoles under field conditions. We also tested to see whether the presence of tadpoles (and/or simply the suppression cue) in a waterbody deters adult cane toads from laying their eggs within that water body. Our study was designed to directly inform cane toad control efforts in Australia.

## 2 MATERIALS AND METHODS

### 2.1 Experimental protocols

#### 2.1.1 Laboratory trials on suppressor density

Adult cane toads were collected by hand from Middle Point, Northern Territory (−12.579602, 131.313863) and brought back to a nearby laboratory where they were injected with leuprorelin acetate (Lucrin, Abbott Australasia) to induce breeding (see Hayes, Crossland, Hagman, Capon, & Shine, 2009 for detailed methods). Newly laid clutches of eggs were placed in individual 18 L tubs in unchlorinated water at a constant temperature (26°C) and aerated until they reached developmental stage 18 (Gosner, 1960).

Four circular pools (2,200 mm diameter) were filled with 1,800 L of unchlorinated water. An enclosed mesh net (700 × 400 × 300 mm, mesh size 1 × 1 mm) was placed into the center of each pool and secured so that the top of the enclosure was 5 cm above the water surface. Twelve hours before the experiment began, each net was filled with 300, 30, 3, or zero (control) live cane toad tadpoles (stage 28–34) from an older Middle Point clutch.

When the experimental clutch reached Gosner stage 18, 10 of the hatchlings were placed in each of 32 enclosed circular plastic containers (diameter 76 mm and height 24 mm) with mesh sides to allow water flow‐through. Eight of these containers were then placed into each of the four pools and weighted so that they sat at the bottom of the water column. Four of the containers were positioned at the edge of the pool (75 cm from the tadpole net, = “far”) and four were placed beside the net (“near”). The containers were left in the pools for 48 hr (until hatchlings reached stage 25) to allow exposure to cues from the netted tadpoles. They were then removed, and five of the now free‐swimming tadpoles from each experimental container were randomly selected and placed together into a clean 1 L plastic container filled with 750 ml of fresh unchlorinated water. The tadpoles were fed crushed algal wafers daily, and water was changed every second day. Ten days later, the tadpoles were weighed and their developmental stages recorded. We ran this experiment three times, using three different clutches of hatchlings, and exposing them to three different clutches of tadpoles.

#### 2.1.2 Field and laboratory trials on effects of suppression

**FIGURE 1:**
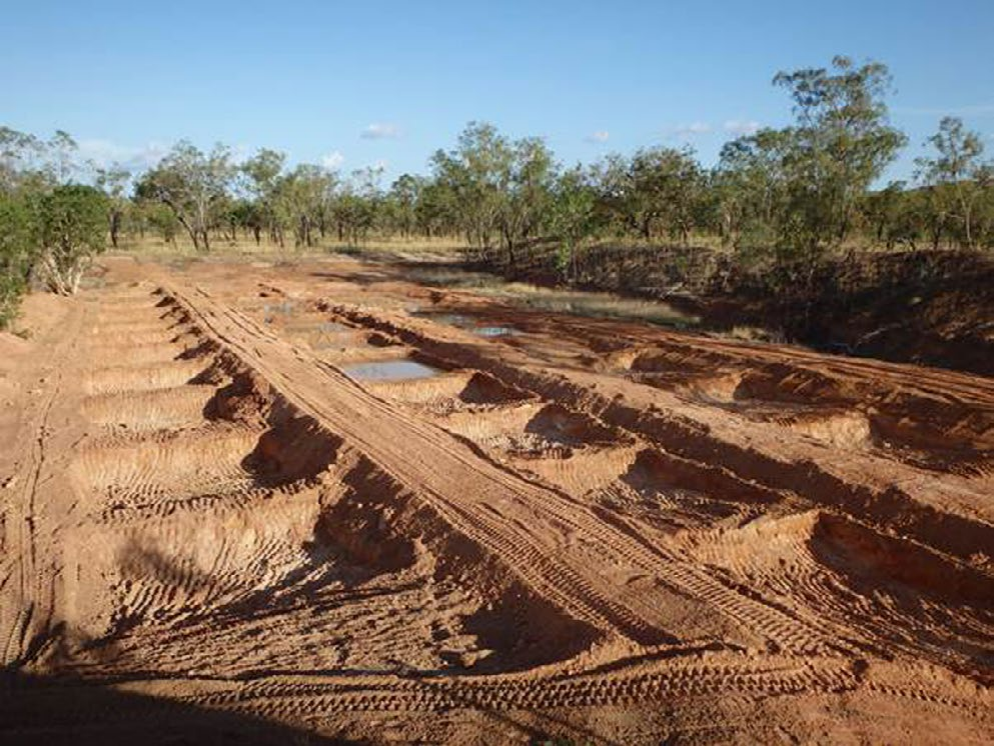
Replicate ponds constructed in a clay‐based depression in bushland 15 km from Kununurra, Western Australia. Eight of these ponds were used for the current study

Two clutches of eggs were obtained from cane toads collected from Kununurra and raised as described above. When the tadpoles reached stage 18, 10 hatchlings were placed in each of eight enclosed circular plastic containers with mesh sides (diameter 76 mm and height 24 mm), and this was repeated for each clutch. For the field trials, two of these containers (both containing hatchlings from the same clutch) were then placed into each of the eight ponds (four ponds per clutch) and weighted to sit at the bottom of the water column (20 cm deep). Eight replicate ponds (5 × 4 m, 1 m depth at deepest end, gradient to 0 m at opposite end) were dug 2 m apart, in a clay‐based depression in bushland 15 km from Kununurra, Western Australia (−15.827949, 128.856982; Figure 1). The ponds were lined with plastic sheeting (100 μm thick) to help retain water, and covered with 20 mm of natural sediment and 28 L of benthic leaf litter sourced from a nearby waterbody. Ponds were each filled with 7,500 L of water, sourced from the local Lake Kununurra (from an area where toads do not breed), and given 48 hr to settle. An enclosed mesh net (400 × 300 × 300 mm, mesh size 1 × 1 mm) was placed into each pond and secured so that the top of the enclosure was 5 cm above the water surface. Twelve hours before the experiment began, half of the nets were filled with 30 cane toad tadpoles (Gosner stage 28–34) field‐caught from two local populations and half of the nets were left empty (controls). This suppression treatment equaled a density of 0.004 tadpoles/L, falling between the “3 tadpole” (0.002 tadpoles/L) and “30 tadpole” (0.02 tadpoles/L) treatments in the previous laboratory experiment.

For the laboratory trials, another 10 hatchlings were placed into each of 10 plastic aquaria (1 L) containing 750 ml of unchlorinated water, and this was repeated for each clutch. These were assigned to either a “suppression” or “control” treatment. We placed a flyscreen mesh enclosure (60 × 40 × 30 mm, mesh holes 1 × 1 mm) into each aquarium and added two live cane toad tadpoles (one from each of the wild populations used for the pond enclosures) to the container for each of the “suppression” aquaria. In “control” aquaria, the mesh container remained empty. The aquaria were kept in the laboratory at 30°C.

The pond and laboratory treatments were both left for 48 hr (until the hatchlings reached stage 25, when they become capable of swimming) to allow exposure to tadpole‐derived cues, after which they were removed. From each container, five of the now free‐swimming tadpoles were randomly selected and placed into a clean 1 L plastic container filled with 750 ml of fresh unchlorinated water. The tadpoles were fed crushed algal wafers daily, and water was changed every second day. Ten days later, tadpoles were weighed and their developmental stages recorded. They were then returned to the laboratory and raised until they either died or reached metamorphosis. If metamorphosis was reached, the days taken to reach metamorphosis and the size of the metamorph at emergence were recorded.

This experiment was repeated with a further two clutches, allowing enough time for the previous suppression cues to no longer be present in the ponds (Clarke et al., 2015, 2016), and using different suppressor tadpoles. Although measurements were taken at 10 days growth for all clutches tested, time constraints meant that only the first two clutches were run to metamorphosis.

#### 2.1.3 Trials of oviposition behavior

We erected walls of plastic sheeting (500 mm high) across the middle of each of the ponds, to divide each pond evenly into two. Each half of the original pond held 2,500 L, with no water flow between the two sides but a common bank. Twelve hours before the experiment began, we placed a mesh enclosure (200 × 200 × 150 mm) into the middle of each half‐pond. One side was randomly allocated to the “suppression” treatment and the other side left as a control. In the suppression sides, 30 tadpoles (stage 28–34) from a mixture of two wild clutches were placed into the mesh enclosure. Fences (600 mm tall) around each pond (enclosing both of the half‐ponds as a single unit) excluded any wild cane toads.

Adult cane toads were collected by hand from around Kununurra and were injected with leuprorelin acetate (Lucrin, Abbott Australasia) to induce breeding. One female and two males were then placed inside the fence of each pond and left overnight. The next morning we collected any clutches laid in the ponds and recorded the side (half‐pond) in which they had been laid. If no eggs had been laid, the replicate was removed from the data set. This experiment was repeated (with fresh adult toads and fresh suppressor tadpoles) until 10 clutches had been laid.

We then repeated the experiment but instead of restraining the suppression tadpoles (“enclosed suppression”), the mesh enclosures were removed and suppression tadpoles were placed directly into the pond, allowing them to swim freely (“non‐enclosed suppression”). The control side of the pond contained no tadpoles. This experiment was repeated with fresh toads and fresh suppressor tadpoles until seven clutches had been laid (the work was then terminated because of unsuitable weather).

### 2.2 Statistical analysis

Data were normally distributed, so we used parametric tests.

#### 2.2.1 Laboratory trials on suppressor density

The position of embryos in the pond (near or far) had no effect on mass or stage of tadpoles (ANOVA with location as factor, all *p *> .05), and so these treatments were combined for the analysis. The mass and stage of the five tadpoles raised in each 1 L container were averaged, to create one replicate. To test for an effect of suppressor density on the mass and stage of tadpoles, we ran two individual ANOVAs in SPSS v21 (IBM, Armonk, NY), with density treatment as the factor, and included clutch as a random factor. We then ran Tukey's post hoc tests to determine which treatments contributed to the differences observed.

#### 2.2.2 Field and laboratory trials on effects of suppression

To test for an effect of suppression treatment on survival, mass, and stage, we ran two individual two‐way ANOVAs in JMP v9 (SAS Institute, Cary, NC), with “Suppression treatment” and “Location” as the factors, and including “Clutch” as a random factor. To test for an effect of suppression on the mass of metamorphs and on the days taken for individuals to reach metamorphosis, we ran separate ANOVAs in JMP with “Suppression treatment” and “Location” as the factors and including “Clutch” as a random factor.

#### 2.2.3 Trials of oviposition behavior

To test for an effect of enclosed suppression treatment on the likelihood of toads laying in a waterbody, we ran a binomial test in SPSS comparing the number of times toads laid in the suppression treated side of the pond and the number of times they laid in the control side. We repeated this analysis to test for an effect of nonenclosed suppression treatment on the likelihood of toads laying in a waterbody.

## 3 RESULTS

### 3.1 Laboratory trials on suppressor density

**FIGURE 2:**
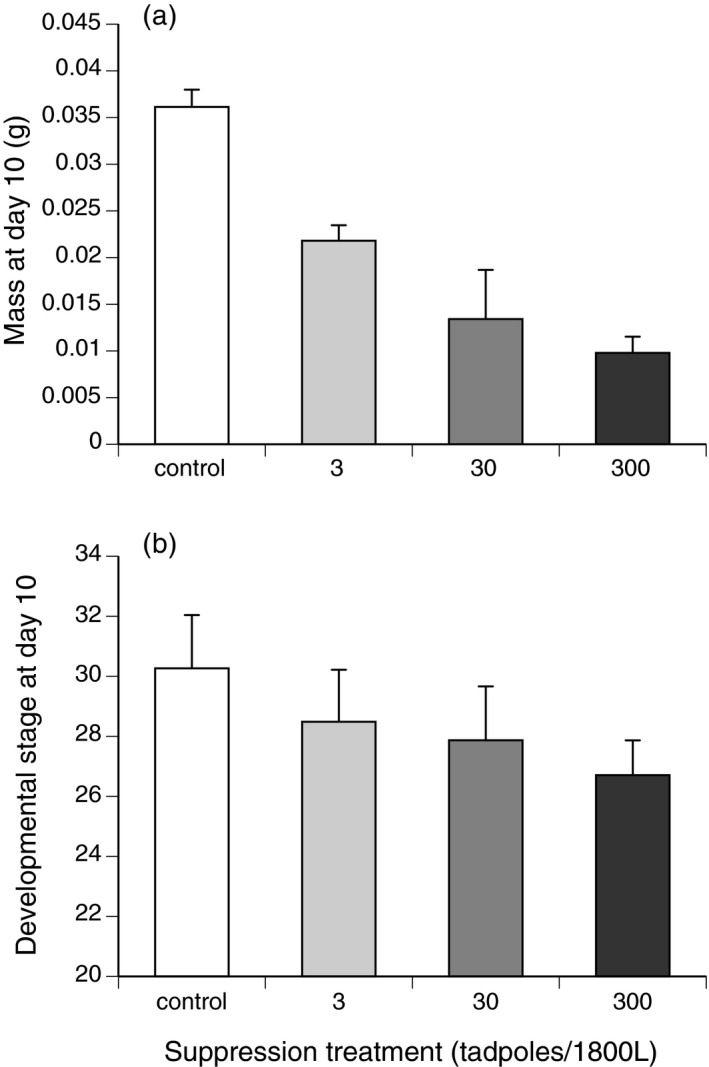
The (a) mass and (b) developmental stage (Gosner, 1960) of cane toad tadpoles (*Rhinella marina*) exposed to suppression treatments, after 10 days of growth. “Control” tadpoles were not exposed to conspecific tadpoles during early development, and supressed tadpoles were exposed to 3, 30, or 300 older conspecifics in 1,800 L of water, for 48 hr during larval development. The graphs show mean values ± *SE*. These data were collected in July 2017, at Middle Point, Northern Territory

Tadpoles that were exposed to the suppression cue weighed less than control tadpoles at day 10, and the density of suppressor tadpoles affected the mean mass of cue‐exposed tadpoles at day 10 (ANOVA, *F*_3,11_ = 104.17, *p* < .001; Figure 2a). Tadpoles exposed to the 300‐suppressor treatment weighed less than tadpoles exposed to the 30‐suppressor treatment that in turn weighed less than tadpoles exposed to the 3‐suppressor treatment (Tukey's post hoc tests, all *p* < .05). The density of suppressor tadpoles also affected the developmental stage of tadpoles at day 10 (ANOVA, *F*_3,11_ = 58.05, *p* < .001; Figure 2b). Control tadpoles were the most developed, followed by those from the 3 and 30 tadpole treatments, followed by those from the 30‐tadpole treatment (Tukey's post hoc tests, all *p* < .05). Exposure to the suppression cue retarded development, with tadpoles exposed to the 300‐suppressor treatment less developed than were tadpoles exposed to the 30‐suppressor or the 3‐suppressor treatment (Figure 2b).

### 3.2 Field and laboratory trials on effects of suppression

**FIGURE 3:**
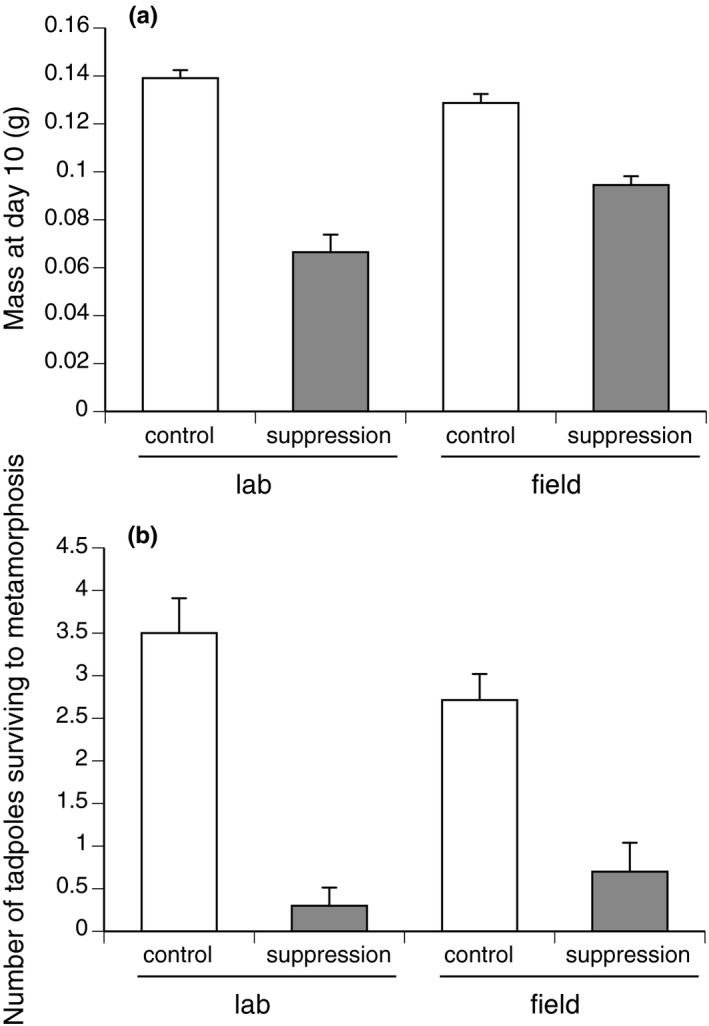
The (a) mass at day 10 and (b) proportion of cane toad tadpoles (*Rhinella marina*) surviving to metamorphosis, after exposure to suppression treatment. “Control” tadpoles were not exposed to conspecific tadpoles, and “suppressed” tadpoles were exposed to older conspecifics for 48 hr during larval development. “Lab” individuals were exposed to two conspecific tadpoles in 750 ml of unchlorinated water and were kept indoors at 30°C. “Field” individuals were exposed to 30 conspecific tadpoles in 7,500 L of water under natural pond conditions and were transferred to laboratory conditions once the exposure period (48 hr) was over. The graphs show mean values ± *SE*. These data were collected in October 2017, at Kununurra, Western Australia

**FIGURE 4:**
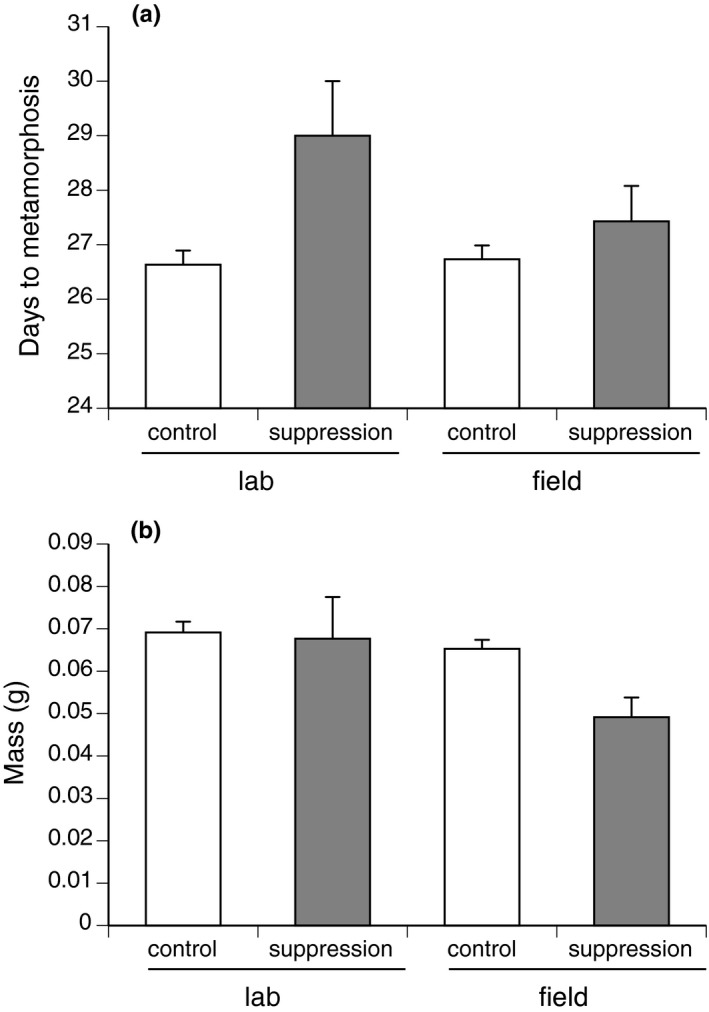
The (a) number of days taken to reach metamorphosis and (b) mass of cane toad metamorphs (*Rhinella marina*), after exposure to suppression treatment. “Control” individuals were not exposed to conspecific tadpoles, and “suppressed” individuals were exposed to older conspecifics for 48 hr during larval development. “Lab” individuals were exposed to two conspecific tadpoles in 750 ml of unchlorinated water and were kept indoors at 30°C. “Field” individuals were exposed to 30 conspecific tadpoles in 7,500 L of water under natural pond conditions and were transferred to laboratory conditions once the exposure period (48 hr) was over. Measurements were taken within 24 hr of metamorphosis (when the tail had been fully absorbed). The graphs show mean values ± *SE*. These data were collected in October 2017, at Kununurra, Western Australia

The mean mass of tadpoles at day 10 was affected by a significant interaction between “Suppression treatment” and “Location” (ANOVA, *F*_3,58_ = 11.19, *p* = .001; Figure 3a). Exposure to the suppression cue reduced tadpole mass more in laboratory trials than in field trials (Figure 4a). However, the reduction in survival rate due to cue exposure (ANOVA *F*_1,27.05_ = 53.97, *p* = .0001) was similar in the laboratory and the field (main effect of laboratory versus field *F*_1,28_ = 0.42, *p* = .52; interaction laboratory–field versus treatment *F*_1,27.05_ = 2.69, *p* = .11; Figure 3b). Individuals exposed to the suppression cue took longer to reach metamorphosis than did nonexposed individuals, both in the laboratory and the field (ANOVA, suppression treatment, *F*_1,65.6_ = 9.26, *p* = .003; main effect laboratory–field *F*_1,65.7_ = 1.37, *p* = .25, interaction laboratory–field versus treatment *F*_1,65.0_ = 2.65, *p* = .11; see Figure 4a). Of the individuals that survived to metamorphosis, body mass at emergence was lower in the field than in the laboratory but with no significant effect of exposure to the suppression cue (ANOVA, location, *F*_1,65.3_ = 4.1, *p* = .047, main effect laboratory–field *F*_1,65.2_ = 2.46, *p* = .12, interaction laboratory–field versus treatment *F*_1,65.0_ = 2.88, *p* = .09; Figure 4b).

### 3.3 Trials of oviposition behavior

**FIGURE 5:**
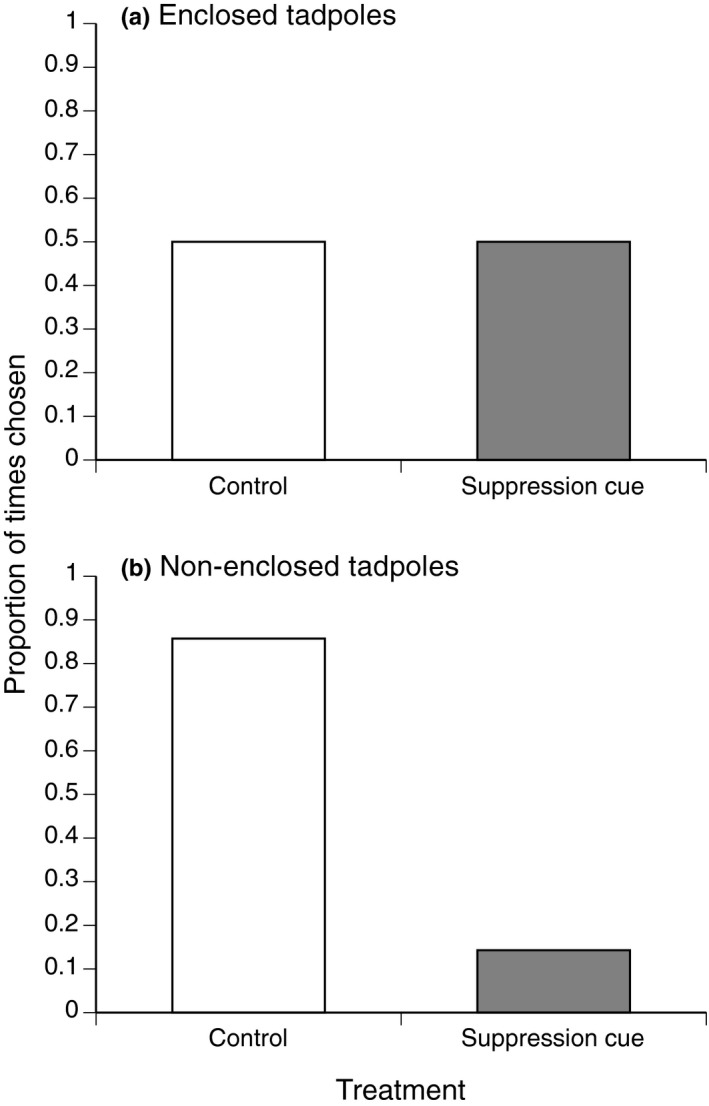
The proportion of times adult cane toads (*Rhinella marina*) laid egg clutches in either the “control” or “suppression cue” side of a pond. Control sections contained no cane toad tapoles, and suppression cue sections contained 30 cane toad tadpoles, either (a) enclosed within a mesh cage (*n* = 10) or (b) free‐swimming (*n* = 7). These data were collected in October 2017, at Kununurra, Western Australia

When suppressor tadpoles were inside a mesh net, adult toads were as likely to lay their eggs in a half‐pond with tadpoles as in controls (5 vs. 5; Figure 5a). However, when suppressor tadpoles were free swimming, more toads laid in control half‐ponds than in those with tadpoles (6 vs. 1, binomial test, *p* = .05; Figure 5b).

## 4 DISCUSSION

Our trials in seminatural waterbodies provided encouraging results. We documented substantial suppression of larvae with low densities of suppressor tadpoles (3 tadpoles per 1,800 L) and found a major reduction in rates of growth, development, and survival of suppressor‐exposed larvae under seminatural field conditions. In nature, smaller‐than‐average tadpoles and metamorphs are highly susceptible to desiccation, and to predation by aquatic and terrestrial invertebrates (e.g., Cabrera‐Guzmán, Crossland, Brown, & Shine, 2013; Child, Phillips, Brown, & Shine, 2008; Ward‐Fear, Brown, & Shine, 2010). Delayed metamorphosis (due to prolongation of the larval phase) also may reduce metamorph survival (e.g., Chelgren, Rosenberg, Heppell, & Gitelman, 2006; Pizzatto, Child, & Shine, 2008). Thus, the reduction in larval viability due to exposure to the suppression cue may be even greater in seminatural and natural waterbodies than in the laboratory. Our experiments also suggested that the presence of tadpoles may discourage oviposition by adult toads, as has been reported also for other species of anurans (Halloy, 2006; Schulte, Krauss, Lötters, Schulze, & Brack, 2015). Low frequencies of reproduction in invasion‐front cane toads (Hudson, Phillips, Brown, & Shine, 2015) mean that anthropogenic addition of chemical cues to isolated waterbodies might significantly reduce the viability of eggs later laid into those ponds.

The data from our laboratory study on suppressor‐cue concentrations support and extend the conclusions from previous work. That work, showing that suppression can be induced even at low densities of older toad tadpoles (0.004 tadpoles/L), was conducted in small (1 L) containers, with the cue evenly dispersed (Clarke et al., 2016; but see DeVore, Crossland, & Shine, 2020). Our study simulated a more realistic situation, whereby the suppression cue was allowed to disperse naturally after being created by tadpoles inside a mesh container within a large (1,800 L) waterbody. Additionally, our study extended the range of concentrations tested and found significant suppression even at 0.002 tadpoles/L. The effectiveness of the cue at such low concentrations, and in such a large volume of water, is promising for the real‐life deployment of the suppression cue in natural waterbodies.

Our field trials confirmed that the suppression cue can work successfully under relatively natural conditions. The waterbodies we used, although artificial, were located in bushland where toads breed, and incorporated factors that are absent from the laboratory setting, and could conceivably modify the impacts of the suppression cue. For example, the ponds we used exhibited strong diel fluctuations in water temperature and light level, and contained aquatic predators such as dragonfly larvae (and thus, waterborne cues from such predators, which can strongly modify developmental trajectories of anuran larvae: Relyea, 2002). The broad attributes of our constructed ponds (size, depth, and exposure to solar radiation) fell well within the range of waterbodies used for breeding by cane toads in tropical Australia (e.g., Hagman & Shine, 2006). Importantly, exposure to the suppression cue during embryonic life imposes severe long‐term impacts rather than immediate mortality. A dramatic reduction in population densities of anuran larvae (as would occur if the suppression cue was instantly fatal) advantages any survivors, because of the reduction in intraspecific competition for food (Alford, 1999; Crossland, Haramura, Salim, Capon, & Shine, 2012). The less intense but more prolonged impact of the suppression cue improves its suitability for toad control, because it means that the few metamorphs surviving after exposure are no larger than conspecifics from control treatments.

Treatment with the suppression cue also prolonged the time taken for tadpoles to metamorphose compared to tadpoles that were not exposed, which can be advantageous for tadpole control overall (McCann, 2018; McCann, Crossland, & Shine, 2020). Longer larval life of cane toads is unlikely to impose additional costs to native wildlife, as the tadpole stage is the least toxic stage of the cane toad life cycle, and predation on toad tadpoles is not a major threat to native anurans (Crossland & Alford, 1998; Hayes et al., 2009). By extending the duration of the larval period, the suppression cue lengthens the window of time that any eggs laid in that pond would be suppressed. In consequence, exposure to the suppression cue generates tadpoles that themselves produce additional suppression cue, over a longer period than would otherwise be the case. That effect reduces the frequency with which managers need to intervene to maintain suppression cue levels within any given pond.

Our final experiment explored a different issue: potential cues that adult cane toads use when choosing to oviposit in a specific pond. Given the highly cannibalistic nature of toad larvae (Crossland et al., 2011), as well as the viability reduction of newly laid eggs exposed to chemical cues from older conspecifics (Clarke et al., 2015, 2016; Crossland & Shine, 2011, 2012; present study), we might expect adult toads to refrain from laying eggs in ponds that already contain conspecific larvae. Avoidance of larvae by ovipositing adults has been noted previously in several species of anuran (e.g., *Physalaemus pustulosus*, Dillon & Fiano, 2000; *Pleurodema borellii*, Halloy, 2006). Although our sample size is small, the same avoidance seems to occur in cane toads. We speculated that chemical cues might be the proximate trigger by which reproducing adult toads detect the presence of larvae (as in Schulte et al., 2015), but our small‐scale field experiment challenged this idea. Adult toads readily laid their eggs in ponds containing the suppression cue, but not in ponds containing free‐ranging tadpoles.

We have not identified the reason why adult toads use the direct presence of tadpoles, rather than chemical cues from those tadpoles, as the proximate cue discouraging oviposition. It remains possible that adult toads cannot detect the suppression cue, or that the danger to any newly laid eggs is a function of the population density of older tadpoles (both through direct cannibalism and suppression‐mediated effects: see Figure 2). If tadpole densities are critical, actual encounter rates with tadpoles may provide more reliable evidence of risk levels than chemical cues. Tadpoles are strongly attracted to the exudate of the parotoid glands of adult toads (Crossland et al., 2012), so an adult female toad laying in the water might well attract tadpoles, providing a clear indication of the degree of risk to any offspring spawned in that pond.

The lack of response of adult toads to the suppression cue (i.e., their willingness to spawn despite the presence of that cue) is useful from a control perspective. It would eliminate the need for managers to locate newly laid egg clutches during their brief window of susceptibility to suppression (from being laid to free‐swimming) in order to apply the suppression treatment. Instead, the cue could be dispensed into waterbodies throughout the breeding season, relying on the apparent preparedness of adult toads to lay their eggs in treated ponds, thereby exposing their clutches to the suppression cue.

We are still a long way from using the suppression cue to control cane toad recruitment in nature. Deploying mesh cages with live cane toad tadpoles is unlikely to be feasible and may raise ethical concerns. Lacking any understanding of the chemical nature of the suppression cue, we can only generate it from live cane toad tadpoles—and it appears to degrade within about 6 days (Clarke et al., 2016). Ideally, we need to develop a synthetic mimic of the cue and a method for slow release of the chemical after deployment. This would eliminate the need for the cue to frequently be re‐applied, increasing the efficiency of the overall system. If these obstacles can be overcome, there would be enormous potential for the suppression cue to be applied by members of the public, providing a simple and effective solution to decrease local abundances of cane toads. Public revulsion against the invasive toads provides strong motivation for toad‐control activities, especially if they do not involve the need to collect and kill adult animals (Shine, 2018). Labor‐intensive methods such as the ones explored in this paper cannot provide landscape‐scale control of cane toads, but they may be a valuable part of an integrated management program in small peri‐urban water bodies.

## CONFLICT OF INTEREST

The authors declare no conflict of interest.

## AUTHOR CONTRIBUTIONS

**Samantha McCann:** Conceptualization (equal); data curation (equal); formal analysis (equal); investigation (equal); methodology (equal); writing – original draft (equal); writing – review & editing (equal). **Michael Crossland:** Conceptualization (equal); methodology (equal); project administration (equal); supervision (equal). **Matthew Greenlees:** Conceptualization (equal); investigation (equal); methodology (equal); supervision (equal). **Richard Shine:** Conceptualization (equal); formal analysis (equal); funding acquisition (equal); project administration (equal); resources (equal); supervision (equal); writing – review & editing (equal).

## Data Availability

All data included in this manuscript: Dryad, https://doi.org/10.5061/dryad.bg79cnp8f.
